# Tenofovir for prevention of mother to child transmission of hepatitis B in migrant women in a resource-limited setting on the Thailand-Myanmar border: a commentary on challenges of implementation

**DOI:** 10.1186/s12939-020-01268-3

**Published:** 2020-09-10

**Authors:** M. Bierhoff, M. J. Rijken, W. Yotyingaphiram, M. Pimanpanarak, M. van Vugt, C. Angkurawaranon, F. Nosten, S. Ehrhardt, C. L. Thio, R. McGready

**Affiliations:** 1 Shoklo Malaria Research Unit, Mahidol-Oxford Tropical Medicine Research Unit, Mahidol University, Mae Sot, 63110 Thailand; 2 Division of Infectious Diseases, Academic UMC, University of Amsterdam, Amsterdam, The Netherlands; 3 Department of Obstetrics and Gynaecology, Amsterdam UMC, University of Amsterdam, Amsterdam, the Netherlands; 4 Julius Global Health, The Julius Centre for Health Sciences, University Medical Centre Utrecht, Utrecht, Netherlands; 5 Department of Family Medicine, Faculty of Medicine, Chiang Mai University, Chiang Mai, 50200 Thailand; 6 Centre for Tropical Medicine and Global Health, Nuffield Department of Medicine Research Building, University of Oxford, Oxford, UK; 7 Department of Epidemiology, Johns Hopkins Bloomberg School of Public Health, Baltimore, MD USA; 8 Department of Medicine, Johns Hopkins University School of Medicine, Baltimore, MD USA

**Keywords:** Barriers, Inequality, HBV, Antiviral therapy

## Abstract

**Background:**

The aim of this manuscript is to highlight challenges in the implementation of maternal tenofovir disoproxil fumarate (tenofovir) for prevention of mother to child transmission (PMTCT) of hepatitis B virus (HBV) in resource limited setting. Current preventive strategies in resource-limited settings fail mainly due to prohibitive costs of hepatitis B immunoglobulin (HBIG) and a high proportion of homebirths, meaning both HBIG and hepatitis B birth dose vaccine are not given. A new strategy for PMTCT without the necessity of HBIG, could be daily tenofovir commenced early in gestation. Implementation challenges to early tenofovir for PMTCT can provide insight to elimination strategies of HBV as the burden of disease is high in resource-limited settings.

**Methods:**

Challenges encountered during implementation of a study of tenofovir for PMTCT before 20 weeks gestation in rural and resource-limited areas on the Thailand-Myanmar border were identified informally from trial study logbooks and formally from comments from patients and staff at monthly visits. ClinicalTrials.gov Identifier: NCT02995005.

**Main body:**

During implementation 171 pregnant women were hepatitis B surface antigen (HBsAg) positive by point of-care test over 19 months (May-2018 until Dec-2019). In this resource-limited setting where historically no clinic has provided tenofovir for PMTCT of HBV, information provided by staff resulted in a high uptake of study screening (95.5% (84/88) when offered to pregnant women. False positive point-of-care rapid tests hinder a test and treat policy for HBV and development of improved rapid tests that include HBeAg and/or HBV DNA would increase efficiency. Integrated care of HBV to antenatal care, transport assistance and local agreements to facilitate access, could increase healthcare at this critical stage of the life course. As safe storage of medication in households in resource-limited setting may not be ideal, interactive counseling about this must be a routine part of care.

**Conclusion:**

Despite challenges, results from the study to date suggest tenofovir can be offered to HBV-infected women in resource-limited settings before 20 weeks gestation with a high uptake of screening, high drug accountability and follow-up, with provision of transportation support. This commentary has highlighted practical implementation issues with suggestions for strategies that support the objective of PMTCT and the World Health Organization goal of HBV elimination by 2030.

## Background

Worldwide, 257 million people are infected with hepatitis B virus (HBV) with a mortality comparable to tuberculosis and higher than human immunodeficiency virus (HIV) and malaria [1, 2]. The United Nations Sustainable Development Goal 3.3 aims to combat viral hepatitis by 2030 through interruption of transmission by interventions on blood and injection safety, harm prevention strategies in people who inject drugs, and by prevention of mother to child transmission (PMTCT) [3].

In highly-endemic areas, such as sub-Saharan Africa and South East Asia, MTCT is the most important source of chronic HBV infection with vertical infections increasing the lifetime risk of developing liver fibrosis 5-fold compared with horizontally acquired infection [4, 5]. Hepatitis B immunoglobulin (HBIG) and hepatitis B birth dose (HepB-BD) monovalent vaccine followed by three doses of polyvalent vaccines provided in the expanded programme of immunization (EPI) have been the central strategies in high- and middle-income countries. Unfortunately, the opportunity to provide HBIG and HepB-BD in the first 12 h after birth is thwarted in resource-limited settings (RLS) due to a lack of essential health services especially facility births, a problem for half of the world’s population [6]. Even with perfect implementation of HBIG and vaccination, 8–32% of infants can be infected via MTCT in HBeAg positive mothers [7, 8].

There is evidence from HIV that antivirals are safe and effective in prevention of MTCT in RLS in Africa and Asia [9–12]. Systematic review and meta-analysis report lamivudine, telbivudine and tenofovir disoproxil fumarate (tenofovir) provided in the third trimester can interrupt MTCT of HBV in hepatitis B e antigen (HBeAg) positive women with high HBV DNA > 10^6^ IU/mL [13]. Recent meta-analysis suggests better prevention of MTCT with telbivudine and tenofovir, and by initiation of antivirals before the third trimester [13]. However the reduction of HBV DNA is highly dependent on starting viraemia and weeks of treatment with tenofovir. If HBV DNA is low at the time of delivery, HBIG may not be necessary, but the precise threshold of HBV DNA to forego HBIG is currently unknown [7, 14–16]. There are no HBV PMTCT studies where HBIG was not provided to the infant.

With the hypothesis that treatment with tenofovir commenced before an estimated gestational age (EGA) of 20 weeks will decrease the HBV DNA sufficiently to negate the need for HBIG, a study commenced in an area of high HBV prevalence among migrant and refugee women on the Thailand Myanmar border (ClinicalTrials.gov Identifier: NCT02995005). This commentary describes the challenges in a RLS with introduction of maternal tenofovir for PMTCT of HBV that will help future implementation of this strategy in similar settings.

## Methods

### Study site

The Thailand Myanmar border has a long and complex history of conflict and population movements. Since 1986 Shoklo Malaria Research Unit (SMRU) has provided humanitarian health care for refugees and since 1997 for migrants. Integrated to basic health care, SMRU has also conducted research on health problems of relevance to marginalized border population including antenatal and birthing services. Maternal and Child Health (MCH) services at SMRU are provided 24 h per day and attendance at antenatal care is voluntary with approximately 2500 new migrant pregnancies registered per year.

### Brief description of PMTCT of HBV before the study

Screening pregnant women at the first antenatal visit for HBV has been routine at SMRU since 2012, but maternal antivirals for PMTCT of HBV is not supported by any organization. HIV screening has been conducted since 2001 with treatment currently provided by Myawaddy Government Hospital, Myanmar. In the migrant and refugee population on the Thailand Myanmar border the prevalence of hepatitis B among pregnant women is 6.2–8.3% [17, 18] and < 0.5% for HIV [19].

SMRU encourages all women to deliver with a skilled birth attendant in the clinic, decreasing the proportion of home deliveries to less than 15% for more than a decade which compares to 75% home deliveries in rural eastern Myanmar [20]. For infants born in SMRU clinics, HepB-BD is provided in the first hours of life [20]. HBIG has been available free of charge for HBeAg positive mothers when humanitarian funding has been sufficient. In most rural clinics in Myanmar, HBIG is not available and the cost of HBIG prohibitive: equivalent to 1 month salary for the average family [21]. At SMRU in 2015, documentation of completion of the HepB-BD was higher than 90%, HBIG 76.5%, and infant EPI-3 doses, 55.1% [20].

Contrary to the border area, Thailand has a high proportion of hospital birth and a strong vaccination program under the Ministry of Public Health with the current Thailand National Guidelines stating that “HBsAg positive pregnant women should be started on tenofovir during pregnancy [22]”; and Myanmar’s first set of guidelines (July 2019) stating: “HBV mono-infected pregnant women who do not meet the criteria for treatment indications should receive tenofovir disoproxil fumarate (tenofovir) 300 mg once a day from 28 week of pregnancy until 3 months after delivery” [23]. These tenofovir policies align with WHO standards that acknowledge usage of antiviral therapy for PMTCT of HBV [2], but there is no practice of them implemented in rural and RLS on either side of the Myanmar-Thailand border.

### Brief description of the study

**Fig. 1:**
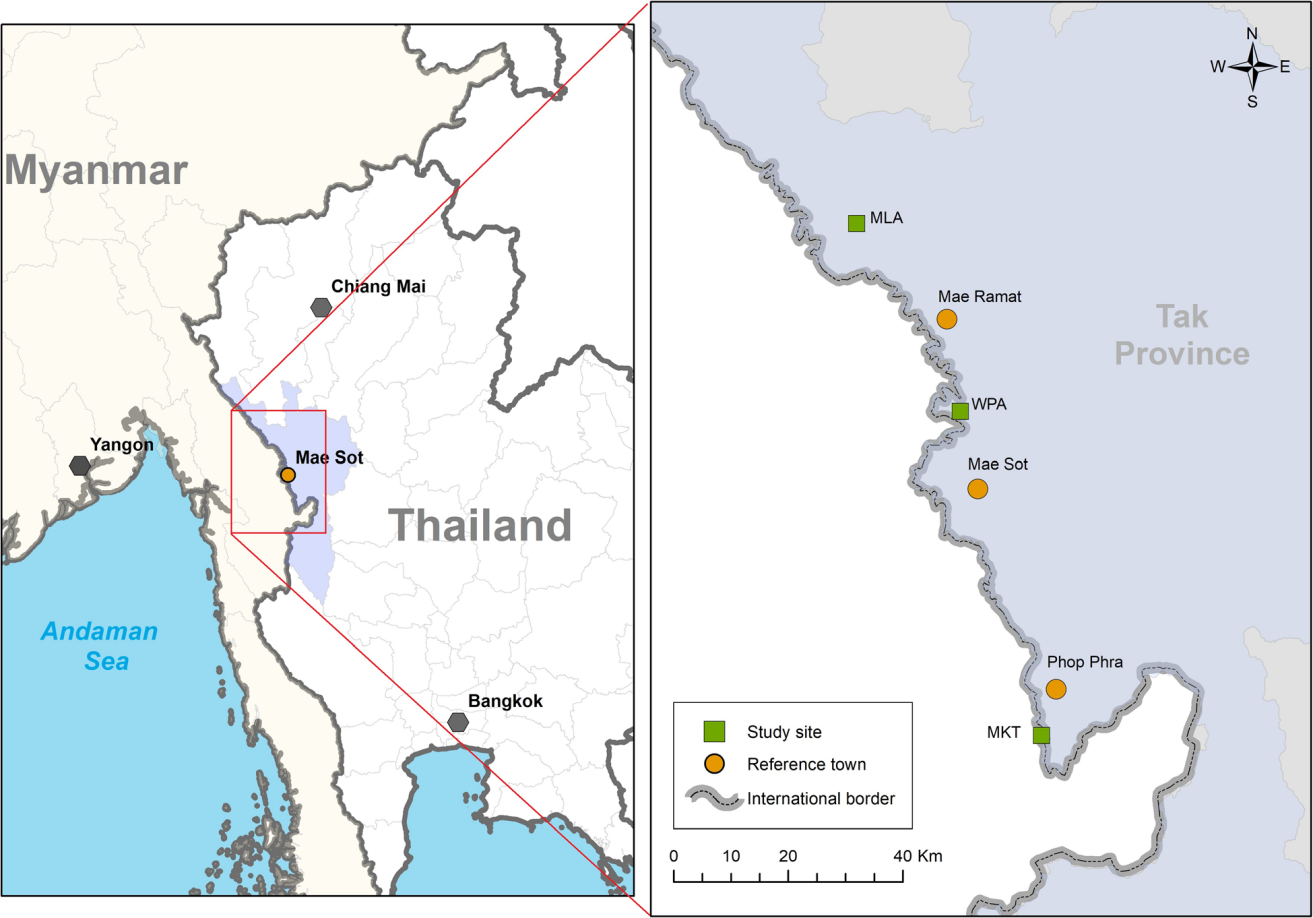
Map of study sites with Mae La Refugee camp (MLA), Wang Pha (WPA) and Mawker Thai (MKT) on the Moei river which demarcates the international border between Myanmar and Thailand [24]

The tenofovir study is a one arm, open label, treatment intervention study, with a sample size of 170 non-Thai pregnant women (16–49 years old) with the primary aim to determine the viral kinetics of HBV DNA reduction in women following maternal tenofovir treatment in pregnancy [24]. Women who meet the inclusion criteria (detectable HBV DNA, ultrasound confirmed viable singleton pregnancy, normal kidney function) can enroll at one of three sites along the Thailand-Myanmar border (Fig. 1). Women are provided with daily tenofovir starting between 12 to < 20 weeks’ EGA until 1 month after delivery, with continued follow-up at months 1, 2, 4 and 6 post-partum. The infant receives immunoprophylaxis including HepB-BD and EPI vaccination; and HBIG in HBeAg positive mothers.

At every pregnancy follow up, alanine aminotransferase (ALT) and creatinine are tested, and participants are asked about adherence using pill count and the ‘Adherence Starts with Knowledge’ questionnaire ASK-12 [25].

### Identification of the challenges

In this commentary, we describe challenges that were encountered during the roll out of the tenofovir study in a RLS. Specific challenges were identified by reviewing the screening and enrolment logbooks to identify barriers to study inclusion of the migrant women that sought ANC care in our clinics. The pharmacy temperature logbooks were reviewed for medication storage, CRFs for comments about medication use, access and adherence by the study participants. Most importantly were the issues that were identified and clarified through discussions between health care workers and participants as they worked through monthly study visits. Missed study visits and missed medication were documented with reasons for these events described and recorded into the CRF. A full description of the study protocol including the consent was published previously [24]. The health care staff involved in the study are part of the community themselves, with an extensive knowledge of the background, culture and language of local women.

### Ethics

This study has ethical approval from Mahidol Oxford Research Unit (FTM ECF-019-06), John Hopkins University (IRB No: 00007432), Chiang Mai University (FAM-2559-04227) and Oxford University (OxTREC Reference: 49–16) and the local Tak Community Advisory Board (TCAB-02/REV/2016).

## Main text

The overall assessment of challenges was based on 171 pregnant women who were HBsAg positive by a point-of-care test (POCT) identified from 31 May 2018 until 31 Dec 2019. The challenges that were encountered were low awareness of HBV treatment for PMTCT, diagnostic limitations, constrained access to healthcare, risk of hepatic flare after cessation of tenofovir, and medication issues (Table 1).

**Table 1:** Challenges and possible solutions for prevention of mother to child transmission of hepatitis B in resource-limited settings

Challenge	Possible Solution
**Low awareness of treatment for HBV prevention**	
There is a gap between government policy and practice for hepatitis B PMTCT in relation to HepB-BD and HBIG in marginalized populations	Implementation of health systems strengthening so policy and practice are aligned e.g. for provision of HepB-BD and HBIG
Knowledge of tenofovir to prevent MTCT of hepatitis B is low	Implementation of a HBV test and treat policy in antenatal care in RLS, alongside other routine first ANC screening tests.
**Diagnostic limitations**	
Misclassification of women with HBV with a single POCT	Accurate POCT or serial or parallel testing of two POCTs
Central laboratory required to identify women at high risk of MTCT	Develop new POCT rapid test for HBeAg and/or HBV DNA using a qualitative test with thresholds as needed
**Constrained access to healthcare**	
Arriving to ANC is constrained by transportation difficulties and costs	Provide integrated care: HBV care coincides with routine antenatal care. Objective assistance e.g. transportation vouchers, if not on foot, to reach ANC can reduce access constraints, at a critical part of the life course.
Checkpoint (Police/Military) fees may increase the stress of antenatal care visits	Agree locally on methods that permit pregnant women passage at checkpoints on their way to antenatal care without additional fees
**Risk of Hepatic flare after cessation of antiviral therapy**	
Hepatic flare risk after tenofovir cessation is uncommon but requires monitoring by ALT	RLS can monitor hepatic flare. New POCT to measure ALT by semi-quantitative measures need to be developed to facilitate this.
	Governmental support under universal health coverage for continuation of TDF treatment for eligible people
**Medication issues**	
Safe storage of medications is difficult in RLS households	Careful counseling helps patients store medication safely
Bioavailability of tenofovir may be reduced by typical tropical weather conditions, including in new off-patent TDF products	Best storage practices require electrical power to maintain medications at recommended temperatures in the tropics and bioavailability for newer TDF products may require confirmation

*ANC* Antenatal care, *ALT* Alanine Aminotransferase, *HBeAg* Hepatitis B e Antigen, *HBIG* Hepatitis B Immunoglobulin, *HBV* Hepatitis B Virus, *HepB-BD* Hepatitis B-Birth dose monovalent vaccine, *HIV* Human Immunodeficiency Virus, *MTCT* Mother to child transmission, *POCT* Point Of Care Test, *RLS* Resource-limited setting, *STI* Sexually Transmitted Infection

### Low awareness of HBV treatment for PMTCT

Although women on the Thailand Myanmar border are receptive to testing for infectious diseases in pregnancy with a near universal uptake of HBV point-of-care-testing (POCT), they still lack awareness of HBV treatment possibilities. In this study, 95.5% (84/88) of the HBsAg positive women with a singleton viable gestation below 20 weeks identified by POCT agreed for further blood investigations. This high uptake suggests a positive response to the concept of treatment of HBV, at least in the context of a study where there is support for study related costs such as transportation.

After general counseling about the infectious diseases that affect pregnancy and after obtaining informed consent, blood was tested for HBV, HIV, syphilis and malaria. Understanding and retention of knowledge from counseling sessions has not been formally tested for HBV in this population. The concern is that disease awareness may be limited by a lack of schooling as two-thirds of women at SMRU ANC did not finish 4th grade [26]. The study is the first in this RLS to introduce the concept that HBV can be treated with drug therapy.

In summary, this study has not changed the already high uptake of HBV screening in pregnancy that was in place before study implementation, but the concept of treatment to prevent HBV transmission to the newborn is new information for local health staff and women. There is a need to measure the knowledge, attitudes and practices in relation to PMTCT of HBV to provide appropriate health messaging to pregnant women and their communities.

### Diagnostic limitations

Identification of women that would benefit from tenofovir is challenging in RLS where the diagnosis relies on HBsAg rapid diagnostic tests and additional testing is not always available. In this study the Pacific Biotech POCT for HBsAg (reported sensitivity > 90% and specificity > 98% [27]) which uses a ‘gold standard’ chemiluminescent microparticle immunoassay had a proportion of false positive of 4/88, 4.5% (95% CI 0.2–8.9). A previous report using the same brand in the same population described a false positive proportion of 3.1% (95% CI 1.7–5.4) [17]. This information is available because the study requires confirmation before treatment. In practice, approaches for HBV could consider using similar diagnostic criteria as for HIV, where two different POCT tests are sufficient to commence treatment, [28] which could lead to a more efficient test and treat policy. However, whether two different HBsAg POCT tests decreases the false positivity rate would need to be determined.

Maternal tenofovir is recommended, at least, for women with a high HBV DNA (> 200,000 IU/mL or HBeAg positive). Of the 84 HBsAg confirmed positive women 73/84 (86.9%) were eligible for the study: 23/73 (31.5%) were HBeAg positive. Women that were HBeAg negative were eligible if they tested positive (50/73, 68.5%) for the presence of HBV DNA (> 85 IU/mL) using a qualitative Polymerase Chain Reaction (PCR) test “HBV DNA assay Fast Track Diagnostics” (Siemens healthineers company). This study detected HBV DNA in HBeAg negative women with an off-site qualitative laboratory test that is expensive and is usually not available in RLSs [29].

In summary, the standard of HBsAg detection by a single POCT is insufficient to confidently identify women with HBsAg and further confirmation is needed.

### Constrained access to healthcare

The ultimate goal of maternal tenofovir treatment for RLSs would be to reduce HBV DNA to undetectable levels to negate the necessity for HBIG at birth [30, 31]. In studies where tenofovir was initiated in third trimester, the HBV DNA was not reduced enough to reach this goal [7, 32]. Commencement of tenofovir in early pregnancy may reduce HBV DNA to undetectable levels before delivery and is the main reason for the current study. However, in RLSs, pregnant women often present after 20 weeks gestational age for antenatal care. As of 31 December 2019, 83/171 (48.5%) of the women in our ANC, who were HBsAg positive by POCT, presented after 20 weeks of pregnancy [18, 33].

**Fig. 2:**
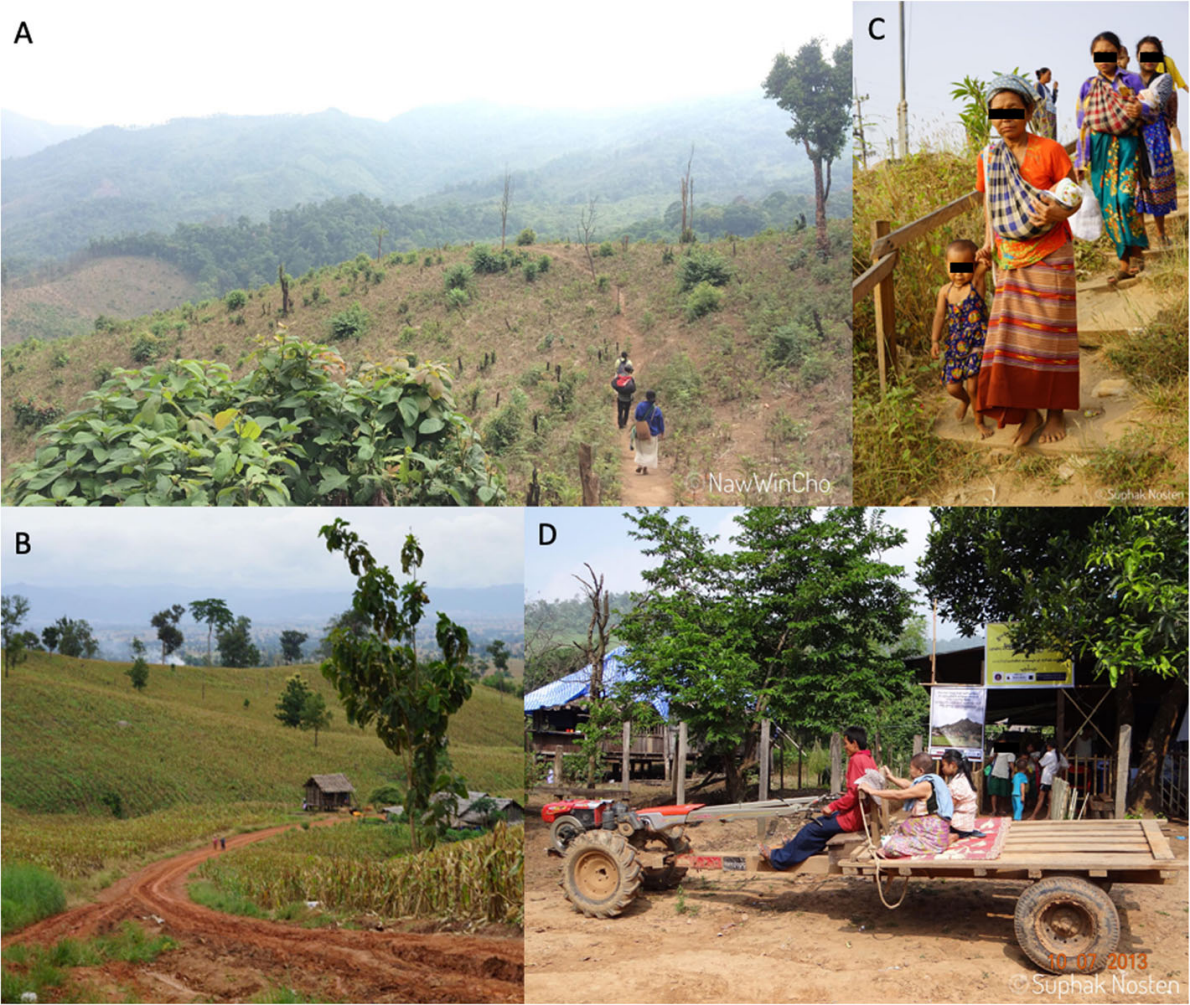
Road leading to the clinic. **a** The distance between a village and the clinic is long and sometimes there is no possibility to use any form of mechanized transportation; **b** Dirt roads might be traversed with a tractor; **c** Most families need to walk many hours with their children to reach the clinic; **d** Tractor used for transportation of patients in Thailand-Myanmar rural border areas

**Fig. 3:**
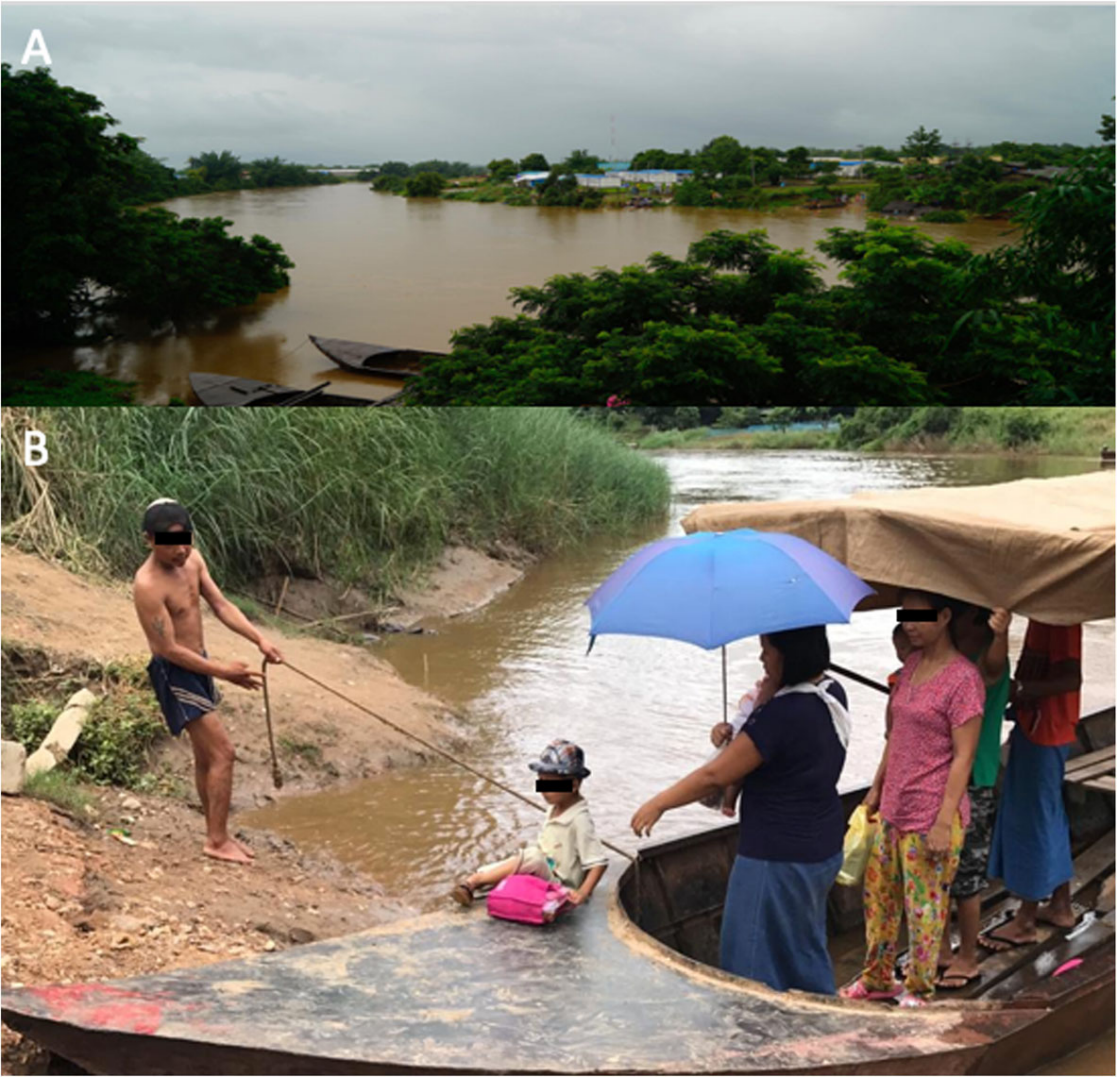
Crossing a river. **a** flooding of the a river during the rainy season is frequent in this mountainous region making it almost impossible to cross which is problematic for scheduled antenatal visits, birth and childhood vaccinations; **b** typical crossing of the river by boat

In rural areas, participants face constraints on access to healthcare. They often live far away from the clinics and this implies that on some occasions these women need to walk for up to 4 h [34] to reach the clinic (Fig. 2). In their travels, most women have to cross one or more river systems, which show large seasonal variations with pronounced flooding (Fig. 3). Of the eight missed appointments in the tenofovir study, five (5/8, 62.5%) were in the rainy season when the rivers became impassable. Travel to the clinic also involves passage through police and/or military checkpoints on both sides of the border. In Thailand, there are documented and undocumented migrant workers with over a quarter of the migrant workers without work permits, or legal status, especially in rural areas. These migrant workers are at risk of being asked for additional fees, or of being arrested at police checkpoints, which can delay presentation with subsequent poorer outcomes. In this study a high follow up proportion of the expected appointments, 395/422 (93.6%) was obtained but women are assisted with transportation. Integrated care allowing pregnant women to complete HBV and ANC care in the same visit will reduce access constraints.

In summary, due to late ANC attendance only one in two women were eligible by gestation for the study. Integrated care alongside reducing access constraints in RLS remains a priority area for general improvement of maternal child health care. Despite difficulties women arrived for their appointments showing that mothers are motivated to overcome difficulties for the health of their pregnancy.

### Risk of flare after cessation of antiviral therapy

After delivery, in RLSs, there is no support to continue HBV antivirals for the mother, should they be needed. Tenofovir treatment ceases 4–12 weeks post-partum and there is a risk for hepatic flare. Hepatic flares are detected using ALT but in most RLSs, ALT is not available on site which can cause a delay in identification of the flare. Most, 90%, of the cases resolves spontaneously, but in rare cases, flares could cause severe disease and even death [35, 36]. This implies that after stopping tenofovir treatment the woman should be able to follow up in a clinic preferably with the ability to measure ALT. In the study, 3/88 (3.4%) women were lost to follow up before the final check for hepatic flare. There was one patient that had a proven, asymptomatic, post-partum hepatic flare that resolved spontaneously after 5 months of additional follow up.

In summary the challenge of detection and management of hepatic flare after cessation of tenofovir post-partum requires careful discourse to ensure the woman remains safe. Flare can be detected and treated in RLS with an offsite laboratory but POCT for ALT would facilitate this.

### Medication issues

**Fig. 4:**
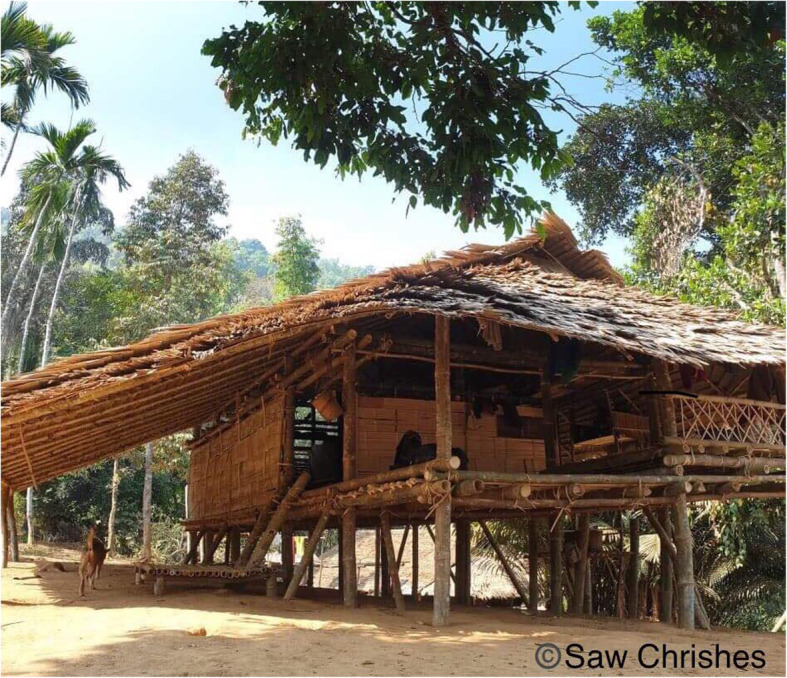
Typical bamboo housing with a leaf roof and bamboo walls and floor

**Fig. 5:**
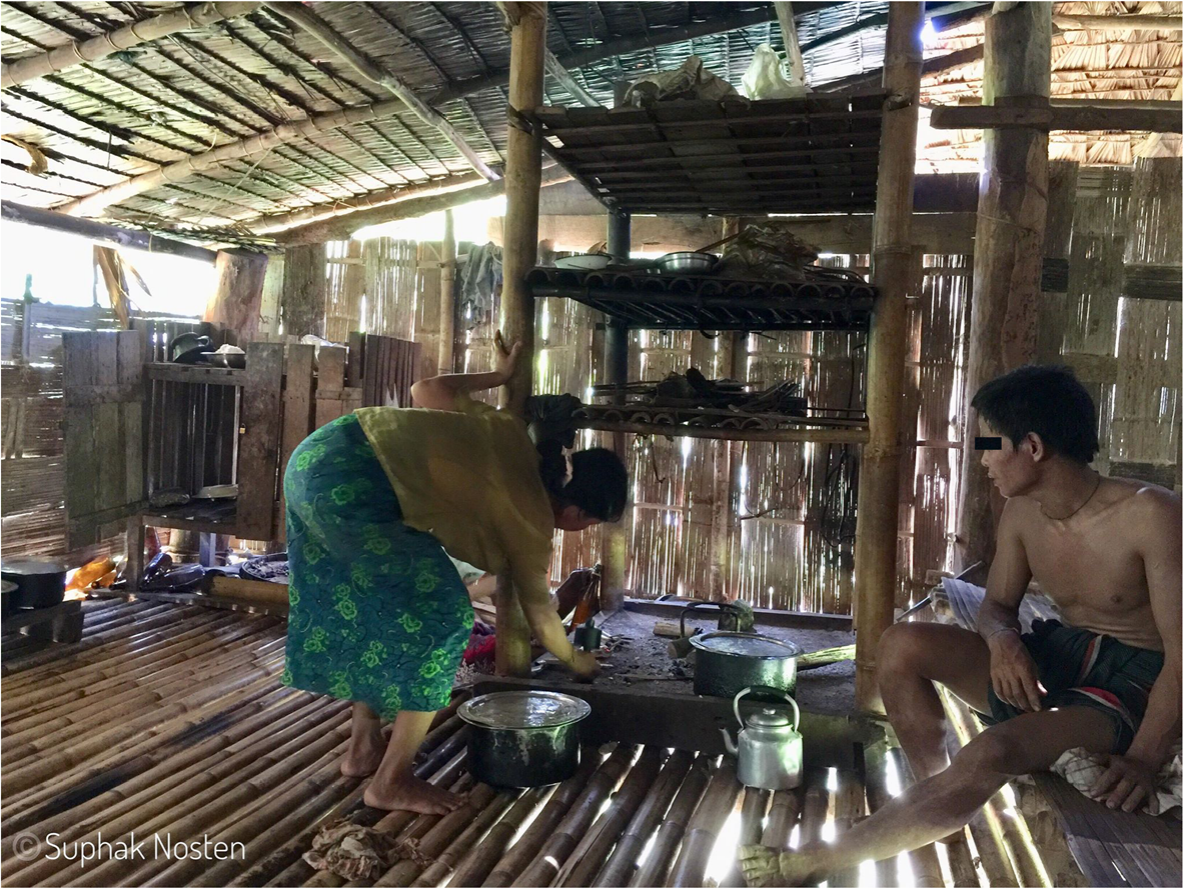
Storage areas for items such as Tenofovir Disoproxil Fumarate. Storage of goods on shelves out of reach of children but above a concrete slab frequently used for cooking increasing the temperature of items on the shelves

Tenofovir should be taken once daily at a dosage of 300 mg. While monthly drug accountability checks have been high (94.6–100% adherence per follow up according to pill count and reviewers opinion), one in ten of the women reported incidents of tablet misplacement: “pills dropped though the *[bamboo]* floor and fell in the mud”, and “children played with the bottle”. The structure of typical households and the places where tablets are normally kept, such as plastic boxes or in an elevated shelf, is normal for families in these areas but may not be ideal or as safe as required (Figs. 4 and 5).

In Mae Sot (Fig. 1), the wet season is oppressive and overcast and the dry season is humid and hot. The highest relative humidity is in August (87%) and the lowest relative humidity in March (61%) [37]. As an example in one of the study sites in 2019: the humidity averaged 76.1% [37] and for 187 days of the year the temperature was ≥30 °C, For 362/365 days (99.2%) humidity and ambient temperature (20-25 °C) were outside the manufacturer’s recommendations: tenofovir should be kept between 20 and 25 degrees Celsius (°C) [38]. In this study medication is maintained at the required temperature via air-conditioning or refrigeration, and the study is monitored closely in line with Good Clinical Practice Guidelines [39]. Participants are supplied with a maximum of 2 months of tenofovir at any one time in air tight resealable bottles of 30 tablets, complete with a silica gel sachet for absorption of moisture that is stored at room temperature as they do not own fridges. In the Gilead patent product information it states that “… tenofovir disoproxil fumarate is prone to decomposition at elevated humidities and temperatures” and the stability is also connected to the pH of the environment [40]. Stability is likely to depend on the formulation of the tenofovir and products that are off patent and cheaper may not maintain the bioavailability or shelf life of the original product manufactured by Gilead.

In summary, most women appeared to manage their medications well but it should not be assumed that households have a safe storage place for medication. Study drugs were well maintained in fridges at the study field site and Gilead reports that Viread bioavailability is not affected by high average humidity and temperature. It is challenging for some women to adhere to good pharmacy practice in the household under normal tropical weather conditions, which may affect bioavailability in new, off-patent products.

## Recommendations

The first step towards prevention of MTCT of hepatitis B is timely vaccination, which can be provided without screening for hepatitis B in pregnancy. This requires a minimum level of government investment in guidelines for policy and finance that supports this practice. Many RLS struggle to reach a high level of facility births, which impedes hepatitis B policy for both HepB-BD and HBIG. The window for prevention is small and is usually passed before home-born children come to the attention of service providers.

As early antenatal care remains a corner-stone to positive birth outcomes overall, not just early tenofovir, settings with late initiation of care can investigate the barriers to this at a local level. Nevertheless, more than 90% of pregnant women in RLS attend antenatal care, and screening for diseases transmitted from mother to child is a global practice. Therefore, adding HBsAg to this routine testing can boost awareness and help identify pregnant women at risk of transmitting to the newborn. Treatment options must be provided and explained as this can support or influence parental decisions of where a mother gives birth [41]. Studies from RLS, and settings where literacy is limited, are required to clarify that antenatal care screening is understood.

Accurate POCTs are crucial to timely implementation of treatment. POCT for HBeAg and/or HBV DNA > 200,000 IU/mL would significantly boost identification of women who will benefit most from tenofovir. Offering treatment during pregnancy presents the highest profile of care for hepatitis B at the community level, just as it does for HIV. For RLS a critical question that requires an answer because health systems differ between resource-high and -limited settings is: Does tenofovir given in early pregnancy reduce the HBV DNA concentration to undetectable levels allowing the elimination of HBIG, which is unaffordable and difficult to maintain in RLS? This will be clarified when the tenofovir study results are analyzed.

## Conclusions

Despite challenges, results from the study to date suggest tenofovir can be offered to HBV-infected women in RLS before 20 weeks gestation with a high uptake of screening, high drug accountability and follow-up, albeit with provision of transportation support related to the study. This commentary reports on a small and local study, but it is clear that mothers in RLS have the interest of their future offspring at heart and will go to serious efforts to obtain good outcomes. Governments and health service providers can take steps towards PMTCT of HBV by policies that are met with finance that ensure practice is harmonized towards the 2016 World Health Organization goal of viral hepatitis elimination by 2030.

## Data Availability

Data sharing is not applicable to this article as no datasets were generated or analysed during the current study.
